# Worldwide Research Trends on Diabetic Foot Ulcers (2004–2020): Suggestions for Researchers

**DOI:** 10.1155/2022/7991031

**Published:** 2022-01-27

**Authors:** Pin Deng, Hongshuo Shi, Xuyue Pan, Huan Liang, Shulong Wang, Junde Wu, Wei Zhang, Fasen Huang, Xiaojie Sun, Hanjie Zhu, Zhaojun Chen

**Affiliations:** ^1^Department of Hand and Foot Surgery, Beijing University of Chinese Medicine Third Affiliated Hospital, Beijing 100029, China; ^2^College of Traditional Chinese Medicine, Shandong University of Traditional Chinese Medicine, Jinan 250000, China; ^3^Orthopaedic Center, Beijing University of Chinese Medicine Third Affiliated Hospital, Beijing 100029, China

## Abstract

**Objectives:**

Diabetic foot ulcer (DFU) is one of the devastating complications of diabetes. It has high mortality and disability rates. The number of research articles on DFUs has increased. This study was designed to explore the global trends and research hotspots of DFUs to benefit researchers in shaping future research directions.

**Methods:**

Literatures relating to DFU from 2004 to 2020 were retrieved from the Science Citation Index Expanded (SCI-expanded) of Web of Science Core Collection (WoSCC). The current status of DFU research (including publications, journals, the performances of relevant countries, institutions, and authors and the research trends and hotspots of DFU) was analyzed with the WoSCC. VOSviewer v1.6.10.0 was utilised for cocitation, coauthorship, cooccurrence analyses, and bibliographic coupling.

**Results:**

A total of 5869 publications on DFUs were retrieved. We performed a longitudinal review of publications over 17 years: 4500 articles and 865 review articles on DFUs published from 2004 to 2020 were analyzed. The total citation was 107,296. The USA (*n* = 1866), England (*n* = 606), and China (*n* = 599) were the three largest contributors. The University of Washington had the greatest number of publications within this time period (*n* = 103), and it had the most cooperative units and was in the core position in all research institutions, followed by the University of Manchester (*n* = 94) and the University of Miami (*n* = 92). Armstrong DG (91/1.69%) and Lavery LA (55/1.19%) should be regarded as scholars who have made outstanding contributions. The top journal with the greatest total link strength was *Diabetes Care*. Analysis showed that the global research hotspots of DFU focused on lower limb amputation, diabetic foot infection, and treatment and management of DFU. Studies on osteomyelitis, wound therapy and management, multidisciplinary integration and mechanism of DFUs, and its related diseases are the research fronts that should be closely watched in the future.

**Conclusions:**

This study revealed the current research status and hotspots in the domain of DFU over the past 17 years, which can help researchers to further pinpoint potential perspectives on hot topics and research frontiers.

## 1. Introduction

Diabetes mellitus is a common chronic disease. Diabetic foot ulcer (DFU) is one of the devastating complications of diabetes. Poor control of diabetes, distal circulation disorder, or neurological impairment are the main causes of foot and lower leg ulcers. Severe cases may require amputation of the toes, part of the foot, or lower leg [1]. More than 85% of foot amputations are caused by DFU [2]. Syed et al. pointed out that the average nursing cost of hospitalised patients with DFU is 49.6% higher than that of inpatients with non-DFU-related diabetes [3]. DFU brings serious psychological, physical, and economic burdens to patients, so the International Diabetes Foundation is paying close attention to this disorder [4].

In general, the current status and research hotspots in a field of research are summarised by a literature review, which is easily affected by subjective factors and has some defects. Meanwhile, researchers and healthcare workers have to face the challenge of the continuous emergence and introduction of new technologies and concepts. Hence, a comprehensive analysis of DFUs is necessary and crucial to evaluate the hotspots and current state in the research of DFUs.

Bibliometrics is an interdisciplinary science, which uses mathematical and statistical methods to quantitatively analyze all knowledge carriers, especially for scientific publications [5]. It is widely used to gauge the scholarly impact of any scientific publication [6]. Furthermore, with the help of bibliometrics, the developing qualitative and quantitative trends of research can be predicted by comparing the studies of main authors, nations, journals, and institutes [7]. The goal of this study was to provide a 17-year (2004 to 2020) longitudinal view of the evolution of the scientific literature on DFU using bibliometric methods. This work is expected to benefit researchers in shaping future research directions and encourage more significant research.

## 2. Methods

### 2.1. Data Sources

A large number of databases can meet the needs of evaluation studies at the global level [8–10]. However, the SCI-expanded of WoSCC database developed by Thomson Scientific was selected for this analysis, which covers lots of international scientific journals with the highest impact and quality, providing a comprehensive, standardised set of data for export. The WoSCC has been widely used in academia [11].

### 2.2. Search Strategy

The first article on DFU was published in 2004, so the timespan for the retrieval of the literature was from 2004 to 2020 to explore the global trends and hotspots in DFU research. In the first step of bibliometric literature analysis, the search term “diabetic foot ulcer” was used in the WoSCC database and retrieved 5869 documents. As part of the search, several queries in succession were conducted, such as using “diabetic foot ulcer∗ OR diabetic foot wound∗” as the search term. Finally, we used “diabetic foot ulcer” as the retrieval term because it led to almost every relevant study result. Retrieval work was performed on the same day (on 30 June 2021) to avoid changes due to daily updates. Informed consent was not required because these data are secondary data without any personal information.

### 2.3. Data Collection

The information of all identified publications, including title, year of publication, author, affiliations, nationalities, journal, abstract, and keywords, was downloaded from the WoSCC database and then opened through Excel 2016. Two authors independently browsed and extracted data from the qualified publications. The data were further analyzed by http://www.bioinformatics.com.cn/.

### 2.4. Bibliometric Analysis

The basic characteristics of publications were retrieved by the intrinsic function of WoSCC. Taking the time (year) as the independent variable and the number of documents as the dependent variable, the correlation regression analysis between the number of documents and time was carried out via curve fitting.

### 2.5. Visualised Analysis

VOSviewer v1.6.10.0. (Leiden University, Leiden, the Netherlands) is a software tool for building and visualising bibliometric network graphs [12]. In this study, VOSviewer was used for coauthorship, bibliographic coupling, cooccurrence, and cocitation analyses. In the network map created by VOSviewer, different nodes represented various elements, such as country, author, institution, and keyword. The size of the nodes reflected the frequency or number of publications. The connections between nodes represented the relationships such as coauthorship, cocitation, or cooccurrence [13]. Additionally, the colour of the node/lines represented different clusters or years.

## 3. Results

### 3.1. Analysis of Global Publications

**Figure 1:**
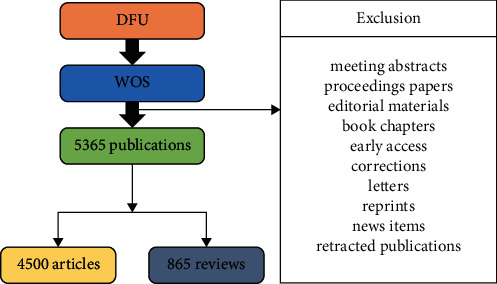
Retrieval strategy and number of publications.

**Figure 2:**
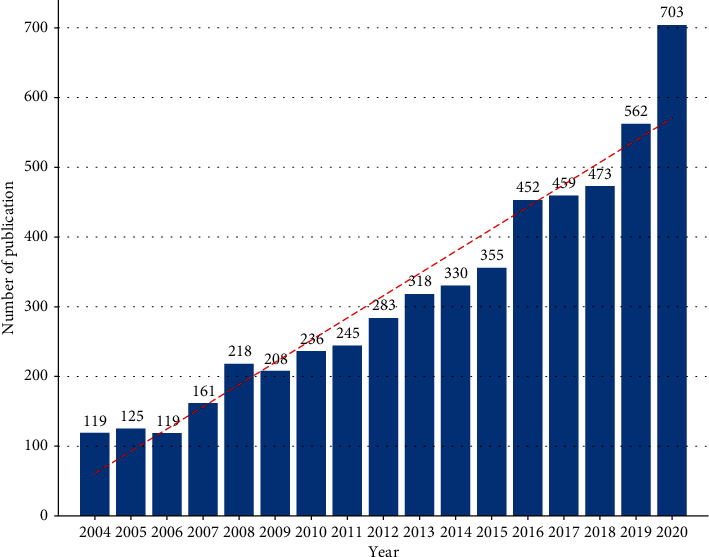
Annual number of publications on DFUs from 2004 to 2020.

A total of 5869 publications were researched after performing the search criteria; 4500 articles and 865 reviews were analyzed. The total citation number for all publications in the field of DFU was 107,296, and the citation number and percent for the USA were 50308 and 46.88%, respectively. Almost all the obtained articles were in English. The retrieval strategy and number of publications are shown in Figure 1. The publishing years of DFU research were divided into three stages: 2004–2009, 2010–2015, and 2016–2020. There were significant differences in the number of documents published from 2004 to 2020 (*P* < 0.0001). The annual amount of publications was significantly correlated with the publication year, and the correlation coefficient *R*^2^ reached 0.9232. The number of publications increased from 119 in 2004 to 703 in 2020. In addition, most of the research was published in 2020 (Figure 2).

**Figure 3:**
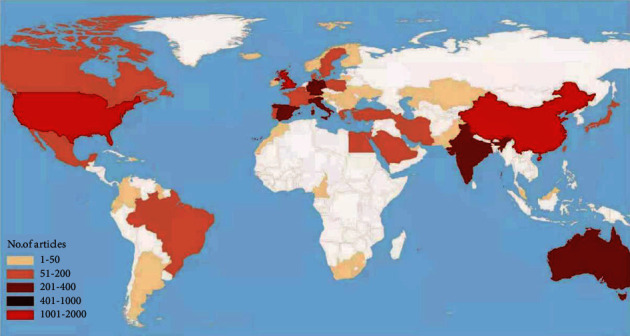
Geographical distribution of DFU-related research articles, 2004–2020.

A total of 100 countries and regions have published related articles/reviews. The geographical distribution of the total number of articles on DFU research from all the countries and regions is shown in Figure 3. Three-quarters of the 5869 articles came from the top eight countries. The USA published the most papers, followed by China, England, Germany, Italy, France, Canada, and Sweden.

Publications are distributed around the world, although output is low in several areas. The countries with the greatest contributions in DFU research are presented in Figure 3. Amongst them, the USA contributed to the most publications (*n* = 1866), followed by England (*n* = 606), China (*n* = 599), and Germany (*n* = 336).

**Figure 4:**
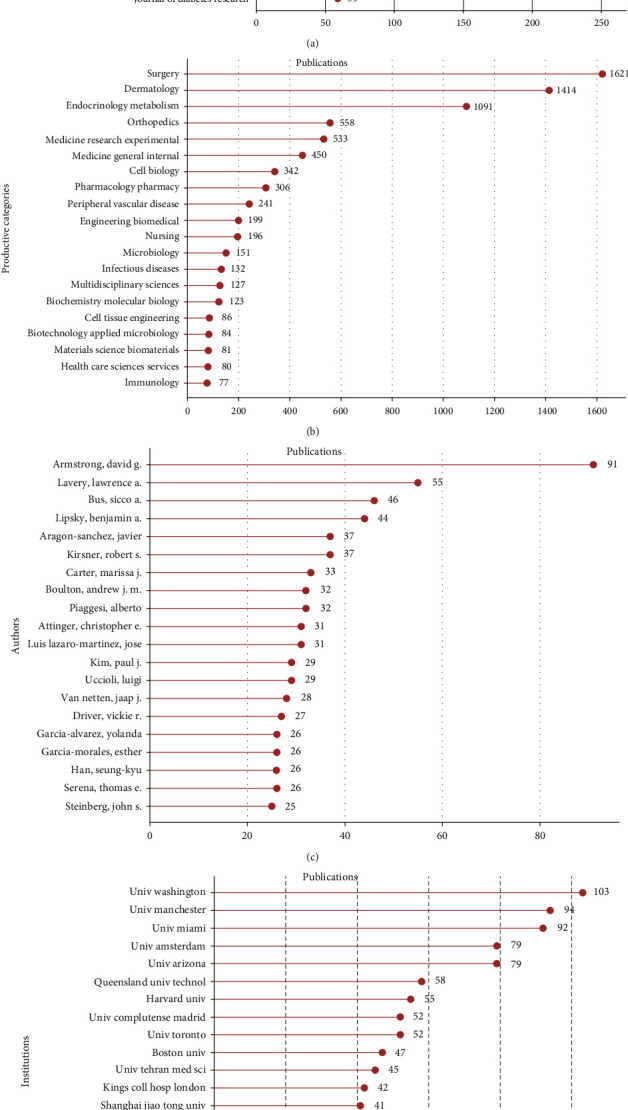
(a) Top 20 journals of publications related to DFU research. (b) The top 20 research productive categories and the number of publications in each category. (c) The top 20 authors of publications. (d) The top 20 institutions with the highest impact and the number of publications for each institution.

*The International Journal of Lower Extremity Wounds (IJLEW)* published 254 articles/reviews, outranking other journals with the most publications. The *International Wound Journal (IWJ)* ranked second, with 235 publications. In addition, there were 234 articles/reviews in the *Journal of Wound Care (JWC)*, 195 in the *Wound Repair and Regeneration*, and 174 in *Wounds A Compendium of Clinical Research and Practice (Wounds)* on the DFU field. The top 20 journals with the most publications are shown in Figure 4(a). The top 10 contributory journals are listed in Table 1.

Articles listed in Table 2 were in descending order of the total number of citations. The top 10 most cited articles were published between 2011 and 2017. Two articles have been cited more than 500 times, published in the *New England Journal of Medicine* (Impact Factor (IF) = 74.69, title: *Diabetic Foot Ulcers and Their Recurrence*, type of study: review, 674 citations) and *Advances in Wound Care* (IF = 4.73, title: *Challenges in the Treatment of Chronic Wounds*, type of study: review, 559 citations).

**Figure 5:**
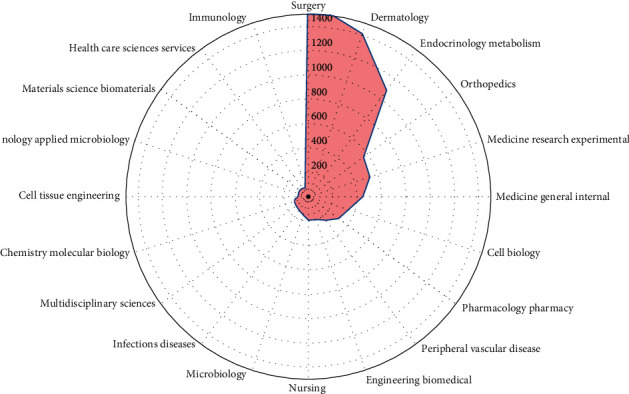
Radar map of the top 20 research productive categories. Note: the radar map was drawn by a bioinformatics online tool (http://www.bioinformatics.com.cn/).

The top 20 productive categories relevant to DFU are presented in Figures 4(b) and 5. The most prevalent categories of research were surgery (1621 papers), dermatology (1414 papers), endocrinology metabolism (1091 papers), orthopaedics (558 papers), and medicine research experimental (533 papers). In terms of research areas, surgery accounted for the largest proportion of publications, whereas immunology accounted for the smallest proportion of publications.

The top 20 authors with the greatest number of publications are presented in Figure 4(c), who have published a total of 711 articles/reviews. David G. Armstrong from the USA outranked other authors with publications of 91 papers, followed by Lawrence A. Lavery from the USA with 55 papers and Sicco A. Bus from the Netherlands with 46 papers.

The University of Washington (USA) had the greatest number of publications with 103 papers, followed by the University of Manchester (the UK, 94 papers) and the University of Miami (USA, 92 papers).  Figure 4(d) presents the top 20 institutions with the most publications.

### 3.2. Quality of the Publications of Each Country/Region

**Figure 6:**
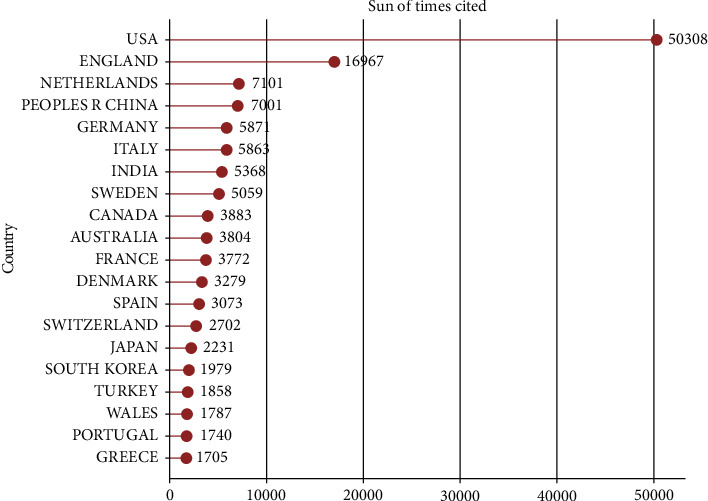
Top 20 countries of total citations.

The USA had the highest total citation frequency (*n* = 50,308), while the UK ranked second (*n* = 16,967), followed by the Netherlands (*n* = 7101), China (*n* = 7001), and Germany (*n* = 5871; Figure 6).

### 3.3. Bibliographic Coupling Analysis

As an accepted method of citation analysis, bibliographic coupling helps establish similar relationships between articles on the basis of the number of references shared by them. When two works mention a common third in their bibliography, bibliographic coupling emerges as an indication of the two works sharing related topics [14].

**Figure 7:**
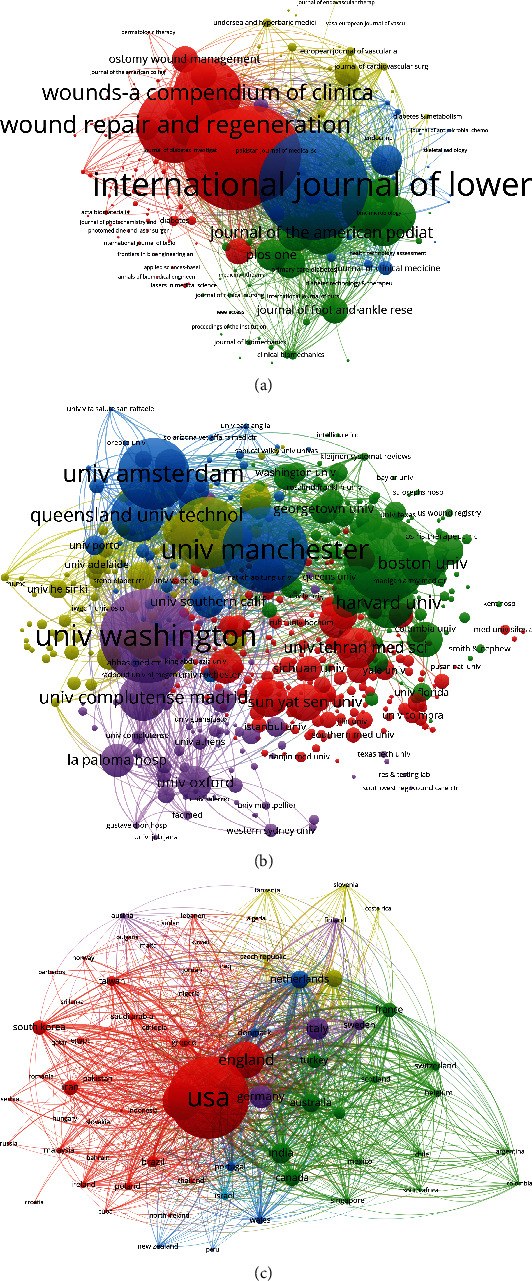
Bibliographic coupling analysis of global research on DFU. (a) Mapping on the 195 included journals in the DFU area. (b) Mapping on the 579 institutions on DFU research. (c) Mapping on the 72 countries in this field. The line between the two nodes indicates the similarity relationship between the corresponding journals/institutions/countries.

VOSviewer was used to analyze the journal names of all publications. As illustrated in Figure 7(a), a total of 195 journals emerged (the minimum number of publications of each journal was over five) in terms of total link strength (TLS). The top five journals with the largest TLS were as follows: International Journal of Lower Extremity Wounds (TLS = 198, 473 times), International Wound Journal (TLS = 168, 517 times), Diabetes-Metabolism Research and Reviews (TLS = 164,713 times), Journal of Wound Care (TLS = 146,647 times), and Wound Repair and Regeneration (TLS = 136,444 times).

Papers originating from 579 institutions were analyzed by VOSviewer, and the minimum number of publications from each institution was over five in the bibliometric map (Figure 7(b)). The top five institutions with the greatest TLS were as follows: University of Washington (TLS = 313,642 times), University of Manchester (TLS = 280,531 times), University of Amsterdam (TLS = 257,075 times), University of Miami (TLS = 256,352 times), and University of Arizona (TLS = 238,037 times).

A total of 72 countries and regions were included (the minimum number of publications from each country or region was over five), and all the publications were analyzed by VOSviewer (Figure 7(c)). The top five countries and regions with the largest TLS were as follows: the USA (TLS = 2,023,416 times), England (TLS = 1,143,981 times), China (TLS = 674,181 times), the Netherlands (TLS = 520,338 times), and Italy (TLS = 491,542 times).

### 3.4. Coauthorship Analysis

Coauthor analysis illustrates projects based on the number of coauthor papers, which is a powerful tool for assessing collaboration trends and identifying leading scientists, countries, and organisations [15].

**Figure 8:**
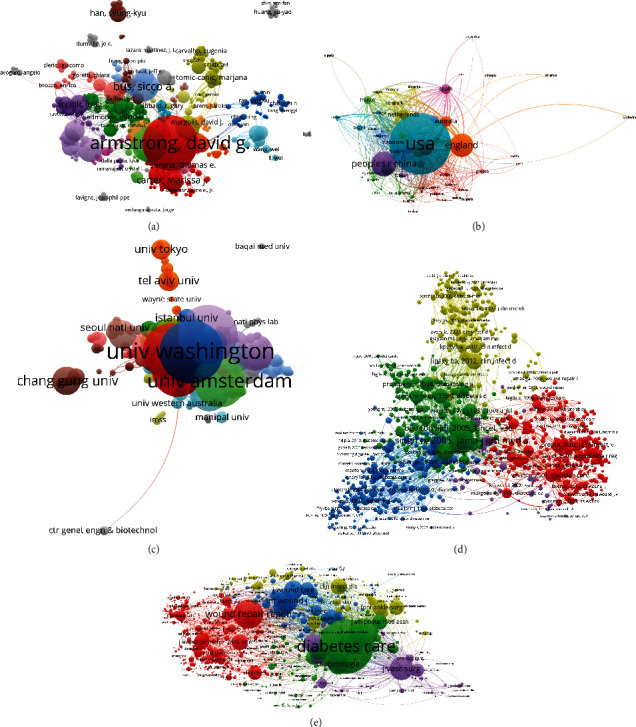
Coauthorship and cocitation analyses of global research on DFU. (a) Mapping of coauthorship analysis amongst 556 identified authors on DFU research. (b) Mapping of 72 identified countries by coauthorship analysis on DFU research. (c) Mapping of coauthorship analysis amongst 579 institutions in the DFU field. (d) Mapping of 1000 included publications by cocitation analysis on DFU research. (e) Mapping on 1000 included journals by cocitation analysis in this field.

As presented in Figure 8(a), a total of 556 authors with a minimum limitation of more than five publications were identified and analyzed with the help of VOSviewer. The top five authors with the greatest TLS were as follows: Armstrong, David G (USA, TLS = 207 times), Lavery, Lawrence A (USA, TLS = 167 times), Uccioli, Luigi (Italy, TLS = 139 times), Piaggesi, Alberto (Italy, TLS = 131 times), and Andluis Lazaro-Martinez, Jose (Spain, TLS = 108 times).

A total of 72 countries and regions with a minimum limitation of more than five publications were identified and analyzed by VOSviewer (Figure 8(b)). The top five countries and regions with the largest TLS were as follows: the USA (TLS = 837 times), England (TLS = 716 times), the Netherlands (TLS = 340 times), Germany (TLS = 312 times), and Australia (TLS = 303 times).

As shown in Figure 8(c), 579 institutions were finally included with a minimum limitation of more than five publications, whose publications were analyzed via VOSviewer. Moreover, University of Washington (USA, TLS = 303 times), University of Arizona (USA, TLS = 224 times), University of Manchester (the UK, TLS = 187 times), University of Miami (USA, TLS = 186 times), and King's College Hospital in London (the UK, TLS = 164 times) were the top five institutions with the greatest TLS.

### 3.5. Cocitation Analysis

Cocitation analysis establishes the relationship of items according to how many times they are cited together, and it is demonstrated as a way to assist in identifying crucial literature for cross-disciplinary ideas [16].

A total of 1000 publications were included (the minimum number of citations for one reference was over 20 times) and analyzed via VOSviewer (Figure 8(d)). The top five publications with the largest TLS were as follows: Singh et al. [17] (TSL = 10,464 times), Ritter [18, 19] (TSL = 7257 times), Armstrong et al. [20] (TSL = 5839 times), Lipsky et al. [21] (TSL = 5530 times), and Pecoraro et al. [22] (TSL = 5478 times).

The journal was included if the minimum number of citations from one source was over 20 times. In total, 1000 journals met the aforementioned criteria (Figure 8(e)). The top five journals with the greatest TLS were illustrated as follows: *Diabetes Care* (TLS = 697,335 times), *Wound repair regen* (TLS = 392,283 times), *J Vasc Surg* (TLS = 308,627 times), *Diabetic med* (TLS = 255,187 times), and the *Int wound J* (TLS = 239,649 times).

### 3.6. Cooccurrence Analysis

Cooccurrence analysis illustrates the relationship of items according to the number of publications where they appear together [23]. Not only could the popular areas and research directions be identified via cooccurrence analysis, but they were also a crucial indicator for monitoring the development of scientific fields and other disciplines. The keywords used more than five times in the included publications were identified and analyzed by VOSviewer.

**Figure 9:**
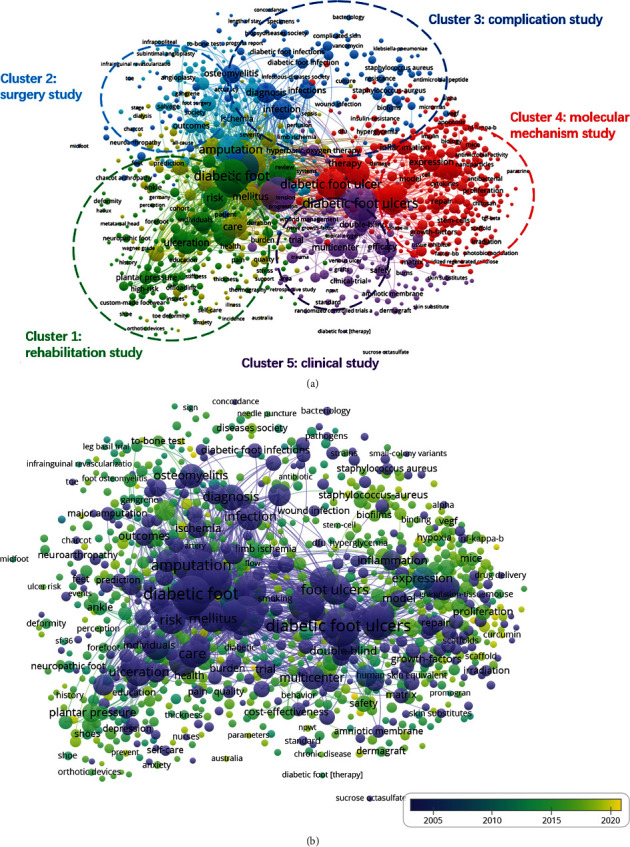
Cooccurrence analysis on DFU research. (a) Mapping of keywords on DFU research; the size of nodes represents the frequency, while the lines between nodes reflect the cooccurrence relationship. A total of 1000 included keywords were divided into five clusters: rehabilitation study (green), surgery study (sea blue), complication study (dark blue), molecular mechanism study (red), and clinical study (purple). (b) Distribution of keywords according to the frequency of appearance. The colour blue indicates the keywords appear earlier, whereas the colour yellow reflects the later occurrence.

As presented in Figure 9(a), 1000 included keywords could be divided into five clusters: “rehabilitation study,” “surgery study,” “complication study,” “molecular mechanism study,” and “clinical study” (Figure 9(a)). The most prominent trends in DFU research were as follows. In the cluster of “rehabilitation study,” the main keywords were diabetic foot ulcer, foot ulcer, and prevention. As for the “surgery study” cluster, the primary keywords were follow-up, amputation, limb salvage, and vascular surgery. In the “complication study” cluster, the most used keywords were ulcers, infection, osteomyelitis, diagnosis, and treatment. As for the “molecular mechanism study” cluster, the prominent keywords were wound healing, inflammation, endothelial growth factor, and expression. In the cluster of “clinical study,” the frequently used keywords were efficacy, multicentre, double-blind, and randomized controlled trial.

In Figure 9(b), different colours were applied by VOSviewer for each keyword based on the mean times they appeared in all included publications. The colour blue indicates the keywords that appear earlier, whereas the colour yellow reflects the later occurrence. As shown in Figure 9(b), a trend of balanced development existed in all the five clusters during the two decades. More researchers focused on the clusters of “rehabilitation study,” “surgery study,” and “molecular mechanism study.” However, recent trends indicated that the two other clusters also underwent different degrees of changes on the study hotspot, which meant a diversified developing trend.

## 4. Discussion

### 4.1. Global Trends in DFU Research

Bibliometric analysis and visual analysis are believed to be effective tools for describing the current status and predicting future directions about the studies of interest. Hence, the present study is aimed at assessing DFU research and further illustrating its potential global trends in terms of publications, institutions, contributing countries, and research directions through bibliometric and visual analyses. The domain of DFU research has undergone tremendous development in the past ten decades. As shown in Figure 2, the number of publications has steadily increased year by year. In addition, most of the research was published in 2020, possibly due to the COVID-19 pandemic. During the outbreak of COVID-19, DFUs have been proved to be a risk factor for patient mortality [24]. An increasing number of clinicians and researchers are concerned about DFUs. A total of 100 countries and regions have published papers in this field, suggesting that studies focusing on DFU research and providing in-depth knowledge of DFU are likely to increase in the coming years.

### 4.2. Quality and Status of Global Publications

The total number of citations reflects the quality and scholarly impact of a country's publications [25]. According to the present study, the USA outranked the other countries, such as the total number of publications and citations, making the largest contribution to global DFU research. The United Kingdom, the Netherlands, and China also contributed with a considerable total citation frequency. Meanwhile, some countries such as Germany, India, and Canada have also played a crucial role when considering their high citation frequencies. Developing countries also made important contributions. For instance, China ranked amongst the top five concerning the total number of publications and citations. Given the high incidence and high disability rates of DFU, the developing countries have made large investments in the domain of DFU research, which may explain this result.

As for journals, the *IJLEW*, *IWJ*, *JWC*, *Wound Repair and Regeneration*, and *Wounds* made great contributions, as they published the most research on DFUs. The following reasons explain why. Firstly, the impact factor of these journals is one of the possible reasons for this finding. But to a greater extent, we think it is the academic orientation and research fields concerned by these journals which are more relevant to researchers' articles that make them more inclined to submit their papers to these journals. To elaborate a bit on that, *IJLEW* is a quarterly publication that publishes original research and overviews of evidence-based diagnostic techniques and methods and surgical and medical treatment of lower limb wounds (such as ulcers and traumatic wounds). *IWJ* has a unique position in helping improve patient care standards and international professional practice. It covers all aspects of the prevention and treatment of wounds and related skin conditions. *JWC* is an authoritative journal of wound care and the main source of all the latest research and clinical information related to tissue vitality. *Wound Repair and Regeneration* is the official journal of The Wound Healing Society, The European Tissue Repair Society, The Australian Wound Management Association, and The Japanese Society for Wound Healing. *Wounds* is the most widely read and peer-reviewed magazine, focusing on wound care and wound research. Whether it is trauma, surgery or nonskin trauma, or DFUs, wound care professionals will turn to *Wounds* to understand the latest research and practice in this evolving field of medicine. In addition, these journals are professional magazines with good popularity and influence, researchers can more easily spread their opinions in the academic circle, so as to discuss and communicate with peers to improve their research level. Finally, these journals have a relatively short review cycle; therefore, researchers are more willing to submit papers to them. According to this trend, the listed journals in Table 1 may continue being the “main channel” for future findings in this domain.

Almost all of the top 20 institutions were from the top five countries with the highest number of publications, half of which were located in the USA, again reflecting the great academic influence of the USA in this field. However, China's institutions were mainly Shanghai Jiaotong University and Sun Yat-sen University, which showed the important role of these first-class institutions in improving a country's academic level. David G. Armstrong, Lawrence A. Lavery, and Sicco A. Bus were the top three authors with the most publications in the research of DFUs. The top 20 authors listed in Figure 4(c) indicate research pioneers that may have a substantial impact on the direction of future research. Hence, to keep abreast of the latest developments in this field, we should attach more importance to their work and give it a relatively high priority. In the present study, bibliographic coupling analysis was performed to explain the similarities between the identified publications in terms of the journal, institution, and country. Bibliographic coupling can occur when two studies share common references in their bibliographies, thereby providing in-depth, direct insight into the connections between these related documents and further explaining how the authors established and used these connections. According to study data, the *IJLEW* was the most closely relevant, and the University of Washington published the most related papers. In terms of countries, the USA outshined other countries and maintained leadership in the field of DFU research (Figure 7(c)). Coauthorship analysis was used to assess the collaboration amongst different authors, countries, and institutions. Research object (author/country/institution) with higher TLS was deemed more likely to collaborate with others. In this study, the results of coauthorship analysis were Armstrong DG, the USA, and the University of Washington. Cocitation analysis was performed to explain the impact of studies, by counting the number of times they were cited together. In this study, *Diabetes Care* was the most frequently cited journal and could be regarded as a landmark study on DFU research.

### 4.3. Research Focus on DFUs

On the basis of the number of publications with cooccurrence, cooccurrence analysis was performed to assess the relationship between the identified items. In addition, it is thought to be an effective method for predicting future trends and hotspots in research areas of interest [26]. In the present study, we showed a network graph of the cooccurrence relationship by analyzing the keywords of all the included research. Finally, five potential research orientations were summarised as follows: “rehabilitation study,” “surgery study,” “complication study,” “molecular mechanism study,” and “clinical study” (Figure 9(a)). We could further shed light on the developing future trend and hotspots through this network graph. As presented in the cooccurrence map, keywords such as diabetic foot, ulcers, amputation, follow-up, wound healing, management, and expression were highlighted with large icons, and they were almost evenly distributed in the orientations of “surgery study,” “rehabilitation study,” “complication study,” and “molecular mechanism study.” Therefore, the input and requirement of high-quality research in these five research directions are necessary.

An overlay visual map is similar to the cooccurrence graph. Items are marked with different colours based on the average time of keyword appearance [25]. It allows for direct monitoring of the progress of research and prediction of future hot topics. As shown in Figure 9(b), the different colours indicate the relevant year of publication. From the results, “molecular mechanism study” accounted for large proportions for the colours green and yellow, which suggested that more studies focused on the research of molecular mechanism study of DFUs after 2015. Nevertheless, in each of these five clusters, nodes of various colours (from purple to yellow) could be found in considerable density, implying a tendency for a balanced development of each of these five research directions in the last decade. Additionally, each direction itself experienced changes in research hotspots, which showed a diversified development trend.

### 4.4. Strength and Limitations

Compared with the existing DFU bibliometric research [27], the time span of the literature we included was longer (17 years), and the number of studies included was more (5365), so we analyzed the fitting relationship between the annual number of documents published in this field and the year. In addition, we supplemented our work with other contents. The number of publications of institutions and journals in the domain of DFUs was analyzed, and the analysis of highly cited papers, cocitation, coauthorship, cooccurrence, and bibliographic coupling was performed.

For a better overall grasp of the current status and future trends of DFU research, bibliometric and visual analyses were carried out to determine hotspots and collaborations amongst different countries, institutions, and authors. Nevertheless, this study will inevitably have some limitations. Firstly, although the included publications were sufficient to reflect the current status, we retrieved data from the WoSCC database only. Therefore, we may have left out some publications because of the database bias. Secondly, the literature we analyzed contained only English literature and no literature in other languages, so some bias may exist [28]. Thirdly, when considering the same abbreviation or different expressions for some authors and keywords, bias may remain. Therefore, there could be discrepancies between our bibliometric analyses and real-world studies. With the wide attention of researchers and the advance of technology, future research of DFUs may show explosive growth.

## 5. Conclusions

Investigations related to DFU are developing rapidly at this point based on bibliometric analysis. The USA has made significant contributions in this field, establishing its leadership in global DFU research. Research on the wound treatment, management strategy, and molecular biology of DFUs has attracted extensive attention. Studies on the treatment methods and molecular mechanism of DFUs will be the hotspots in the future. Besides, multidisciplinary integration, including medicine, biomechanics, materials science, computer science, epidemiology, and other sciences, is becoming a trend in this field. Our study can help researchers better understand the current research status of DFUs from a macro perspective.

## Figures and Tables

**Table 1:** Top 10 contributory journals.

Journal	Number of articles	IF (2019)	Percent of total papers
International Journal of Lower Extremity Wounds	254	2.05	4.73%
International Wound Journal	235	3.31	4.38%
Journal of Wound Care	234	2.07	4.36%
Wound Repair and Regeneration	195	3.61	3.63%
Wounds A Compendium of Clinical Research and Practice	174	1.20	3.24%
Diabetes Metabolism Research and Reviews	142	3.40	2.64%
Diabetes Care	125	14.4	2.32%
Diabetic Medicine	118	3.25	2.19%
Journal of the American Podiatric Medical Association	111	0.68	2.06%
Diabetes Research and Clinical Practice	97	4.56	1.80%

**Table 2:** Top 10 most cited articles.

Rank	Year	Article	IF(2019)	Total citations	Type of study
1	2017	David G. Armstrong, et al. Diabetic foot ulcers and their recurrence. *New England Journal of Medicine*. 2017, 376 (24): 2367-2375	74.69	674	Review
2	2015	Robert G. Frykberg, et al. Challenges in the treatment of chronic wounds. *Advance in Wound Care.* 2015, 4 (9): 560-582	4.73	559	Review
3	2014	Joseph L. Mills, et al. The Society for Vascular Surgery lower extremity threatened limb classification system: risk stratification based on wound, ischemia, and foot infection (WIfI). *Journal Vascular Surgery.* 2014, 59 (1):220	3.40	498	Review
4	2012	P. T. Sudheesh Kumar, et al. Flexible and microporous chitosan hydrogel/Nano ZnO composite bandages for wound dressing: in vitro and in vivo evaluation. *ACS Applied Materials & Interfaces.2012*, 4 (5): 2618-2629	8.75	460	Basic medical study
5	2012	Brian M. Peters, et al. Polymicrobial interactions: impact on pathogenesis and human disease. *Clinical Microbiology Reviews*. 2012, 25 (1): 193-+	22.55	374	Review
6	2013	Liane I.F. Moura, et al. Recent advances on the development of wound dressings for diabetic foot ulcer treatment—a review. *Acta Materialia Inc*. 2013, 9 (7):7093-7114	7.24	344	Review
7	2015	Boateng, Joshua, et al. Advanced therapeutic dressings for effective wound healing—a review. *Journal of Pharmaceutical Sciences.* 2015, 104 (11): 3653-3680	2.99	339	Review
8	2017	Pengzi Zhang, et al. Global epidemiology of diabetic foot ulceration: a systematic review and meta-analysis. *Annals of Medicine*. 2017, 49 (2): 106-116	3.24	323	Review
9	2011	Stephen R. Thom, et al. Hyperbaric oxygen – its mechanisms and efficacy. *Plast Reconstr Surgery*. 2011, 127 (1): 131S-141S	4.20	316	Review
10	2011	Debin Lu, et al. Comparison of bone marrow mesenchymal stem cells with bone marrow-derived mononuclear cells for treatment of diabetic critical limb ischemia and foot ulcer: a double-blind, randomized, controlled trial. *Diabetes Research and Clinical Practice*. 2011, 92 (1): 26-36	4.23	275	Clinical research

## Data Availability

The bibliometric research data used to support the findings of this study are available from the corresponding author upon request.

## Abbreviations

DFU: Diabetic foot ulcer
SCI-expanded: Science Citation Index Expanded
WoSCC: Web of Science Core Collection
TLS: Total link strength
IJLEW: International Journal of Lower Extremity Wounds
IWJ: International Wound Journal
JWC: Journal of Wound Care
Wounds: Wounds A Compendium of Clinical Research and Practice
IF: Impact factor.
